# BMP signaling inhibition in *Drosophila* secondary cells remodels the seminal proteome and self and rival ejaculate functions

**DOI:** 10.1073/pnas.1914491116

**Published:** 2019-11-18

**Authors:** Ben R. Hopkins, Irem Sepil, Sarah Bonham, Thomas Miller, Philip D. Charles, Roman Fischer, Benedikt M. Kessler, Clive Wilson, Stuart Wigby

**Affiliations:** ^a^Edward Grey Institute, Department of Zoology, University of Oxford, OX1 3PS Oxford, United Kingdom;; ^b^Target Discovery Institute (TDI) Mass Spectrometry Laboratory, Target Discovery Institute, Nuffield Department of Medicine, University of Oxford, OX3 7BN Oxford, United Kingdom;; ^c^Department of Physiology, Anatomy, and Genetics, University of Oxford, OX1 3QX Oxford, United Kingdom

**Keywords:** reproduction, seminal fluid, sexual selection, sperm competition, sperm

## Abstract

Seminal fluid proteins (SFPs) exert potent effects on male and female fitness. Rapidly evolving and molecularly diverse, they derive from multiple male secretory cells and tissues. In *Drosophila melanogaster*, most SFPs are produced in the accessory glands, which are composed of ∼1,000 fertility-enhancing “main cells” and ∼40 more functionally cryptic “secondary cells.” Inhibition of bone morphogenetic protein (BMP) signaling in secondary cells suppresses secretion, leading to a unique uncoupling of normal female postmating responses to the ejaculate: refractoriness stimulation is impaired, but offspring production is not. Secondary-cell secretions might therefore make highly specific contributions to the seminal proteome and ejaculate function; alternatively, they might regulate more global—but hitherto undiscovered—SFP functions and proteome composition. Here, we present data that support the latter model. We show that in addition to previously reported phenotypes, secondary-cell-specific BMP signaling inhibition compromises sperm storage and increases female sperm use efficiency. It also impacts second male sperm, tending to slow entry into storage and delay ejection. First male paternity is enhanced, which suggests a constraint on ejaculate evolution whereby high female refractoriness and sperm competitiveness are mutually exclusive. Using quantitative proteomics, we reveal changes to the seminal proteome that surprisingly encompass alterations to main-cell–derived proteins, indicating important cross-talk between classes of SFP-secreting cells. Our results demonstrate that ejaculate composition and function emerge from the integrated action of multiple secretory cell types, suggesting that modification to the cellular make-up of seminal-fluid-producing tissues is an important factor in ejaculate evolution.

## Significance

How are ejaculates built? Fertility-enhancing seminal fluid is the product of many different glands and cells, but how the function and composition of seminal fluid emerges from these different elements is poorly resolved. Here, we characterize the contributions of the functionally cryptic *Drosophila* accessory gland secondary cells to ejaculate composition and reproductive outcome. We find that in adults these cells are central to the regulation of the seminal proteome, the promotion of normal sperm behavior, and the induction of many, but not all, postmating responses. Our results illustrate interdependency between glandular cell types, identify constraints in ejaculate functions linked to male reproductive success, and provide insights into the design and production of ejaculates.

Ejaculates are compositionally rich. In addition to sperm, males transfer a mixture of proteins (seminal fluid proteins [SFPs]), lipids, salts, vesicles, and nucleic acids, which together constitute the seminal fluid (1–3). The phenotypic effects of seminal fluid in females are broad, particularly in invertebrates. In various species these effects include increased aggression, reduced sexual receptivity, shifts in dietary preference, conformational changes in the reproductive tract, immunomodulation, and stimulation of offspring production (reviewed in refs. 4–6). A number of SFPs have been further implicated in sperm competition, the process by which sperm from different males compete for fertilizations (7–10). Consequently, seminal fluid represents a critical mediator of male reproductive success (11, 12).

While sperm are always produced in testes, seminal fluid generally comprises products drawn from a number of reproductive tissues (13). These tissues vary considerably in number, cellular make-up, and developmental identity among species, with lineages showing evolutionary patterns of loss, modification, and acquisition (4, 13–15). Why male reproductive systems incorporate this diversity is unclear. It has been suggested that by sequestering SFPs in different cells or glands, males are afforded control over their release and, consequently, afforded spatiotemporal control over their interactions with sperm, the female reproductive tract, and with other SFPs (16). Additionally, functional diversification of tissues and cell types may be required to build specialized parts of the ejaculate, such as mating plugs (17). In either case, activities may be carried out independently between cell types and tissues or there may be cross-talk between them that coordinates global seminal fluid composition. It has been suggested that such cross-talk may be required to drive the sophisticated strategic changes in ejaculate composition observed in relation to sperm competition threat (18). Fundamentally, to understand how ejaculates evolve it is essential that we understand the drivers of diversity in the elements within the male reproductive system, as well as the functional connectivity between them.

**Fig. 1.**
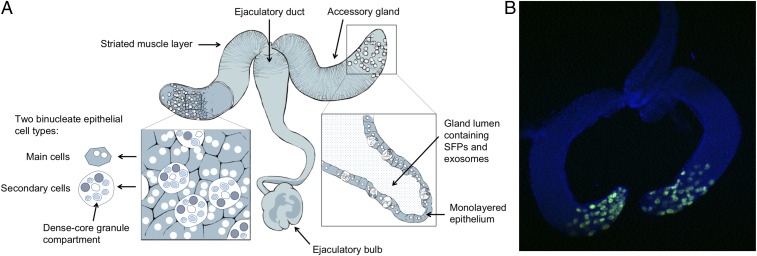
(*A*) The architecture of the *D. melanogaster* male reproductive system. The testes, which branch off from where the 2 lobes of the accessory glands meet, are not shown. Figure adapted from ref. 32. (*B*) Dissected accessory glands from a control (*esg-GAL4* x *w*^*1118*^) male. Secondary cells’ fluorescence derives from *UAS-GFP*_*nls*_. Nuclei stained with DAPI. Image courtesy of Aashika Sekar.

The male reproductive system of *Drosophila melanogaster* consists of testes that produce sperm, and 3 secretory tissues that contribute to the seminal fluid: the paired accessory glands, ejaculatory duct, and ejaculatory bulb (4) (Fig. 1*A*). The majority of the ∼200 SFPs known to be transferred to females are produced and stored in the accessory glands (19). Each of the 2 lobes of the glands is composed of 2 distinct cell types (20). The majority are the ∼1,000 small, binucleate “main cells” (20), which are thought to produce most of the gland’s secretion (21). Accordingly, these cells have been shown to be the sole production site for several highly abundant and functionally important SFPs, including sex peptide (SP), a key driver of postmating changes (22–25). Ablation of main cells leads to failures in the induction of the main female postmating responses: receptivity to remating remains high, and egg production unstimulated (26).

The distal tips of each gland also contain a further subpopulation of ∼40 unusually large “secondary cells” (refs. 20 and 27 and Fig. 1*B*). As with main cells, failures in normal secondary-cell development are associated with defective postmating responses: high receptivity, low fecundity (28, 29). This is partly attributable to glycosylation defects in “SP network” proteins, which are required for the storage and gradual release of sperm-bound SP within the female sperm storage organs—the process through which SP’s effects are extended over several weeks (28). However, targeted suppression of bone morphogenetic protein (BMP) signaling, a pathway that plays crucial and wide-ranging roles in tissue development and maintenance (30), in adult secondary cells has more specific effects. Adult-specific overexpression of Daughters-against-DPP (Dad), a negative regulator of BMP, suppresses the secretion of nanovesicles (“exosomes”) and dense core granules—packages of secretory material that contain high concentrations of signaling molecules—leading to a decoupling of female postmating responses: fecundity is normally stimulated, but sexual receptivity remains high (27, 31, 32). This raises the prospect that BMP signaling in adult secondary cells acts as a highly targeted mediator of reproductive processes. However, we do not know whether the phenotypic effects are restricted to those already identified or whether secondary-cell BMP signaling is a potentially more global regulator of reproduction. This uncertainty also extends to the effects on the seminal proteome: Does suppression of secretion by BMP signaling inhibition in secondary cells cause highly specific changes to the seminal proteome or does it generate more extensive, gland-wide remodelling? In the present study, we use targeted suppression of BMP signaling in adult secondary cells to test between these models at both the functional and proteomic level.

## Results and Discussion

### Sperm Storage Is Compromised in Dad*-*Mated Females.

**Fig. 2.**
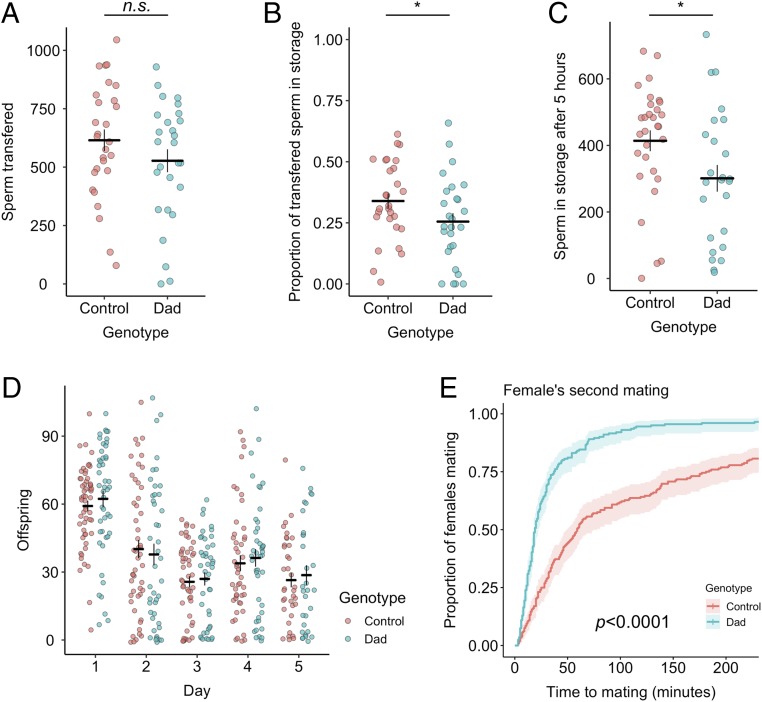
Defective sperm storage and decoupled postmating responses in Dad-mated females. (*A*) The number of sperm present across all regions of the female reproductive tract 25 min after the start of mating, i.e., the number transferred. n_Dad_ = 27, n_control_ = 28. (*B*) The proportion of transferred sperm that has entered into the storage organs (seminal receptacle and spermathecae) at 25 min after the start of mating, n_Dad_ = 27, n_control_ = 28. (*C*) The number of sperm in storage at 5 h after mating, n_Dad_ = 25, n_control_ = 30. (*D*) Daily offspring production, n_Dad_ = 47, n_control_ = 56. (*E*) The latency to remating by Dad- and control-mated females when presented with a second male 24 h later, n_Dad_ = 276, n_control_ = 275. In *A–D,* horizontal bars represent the mean, with vertical bars representing ±1 SE. Data are plotted with horizontal “jitter.” In *E*, confidence intervals are at 95%. *P < 0.05; n.s., not significantly different.

We began by mating virgin females to males who possessed GFP-tagged sperm (33) and who overexpressed the transcriptional repressor of BMP signaling Dad, which suppresses secondary-cell secretion (31) (hereafter “Dad” males), to test whether these secretions are required for normal sperm entry into storage. We found no significant difference between Dad and control males in mating duration (linear model [LM], *F*_1, 110_ = 0.074, *P* = 0.787; *SI Appendix*, Fig. S1) or in the number of sperm transferred (LM, *F*_1, 53_ = 1.700, *P* = 0.198; Fig. 2*A*). The variance in sperm transfer was high for both genotypes, but was consistent with previous reports for *D. melanogaster* (34) and other Diptera (35). The proportion of sperm that initially enters into the storage organs (seminal receptacle and paired spermathecae), and that is ultimately stored (5-h postmating; ref. 33) was significantly lower in Dad-mated females (initial entry at 25 min, generalized linear model [GLM], *F*_1, 53_ = 5.340, *P* = 0.024; Fig. 2*B*; 5-h storage, LM, *F*_1, 53_ = 5.043, *P* = 0.029; Fig. 2*C*). This demonstrates a role for secondary-cell activity in promoting normal sperm storage, which is surprising given that the number of offspring produced by Dad males has previously been shown to be normal (31).

A potential mechanism for reduced storage in Dad-mated females is premature ejection of received sperm (36). However, we found no significant difference in the timing of ejection (Cox proportional hazards [CPH], Likelihood ratio test [*LRT*] = 0.892, *P* = 0.345; *SI Appendix*, Fig. S2), suggesting that secondary-cell activity does not regulate a female’s handling of that male’s ejaculate. Reduced sperm storage in Dad*-*mated females may instead be a consequence of loss of secondary-cell–derived exosomes, the prostate-derived equivalent of which in mammals are known to fuse with sperm and stimulate motility (37). Reduced storage could also arise if secondary-cell BMP signaling inhibition affected SFPs such as the main-cell–produced Acp36DE and/or its associated cofactors, which are known to collectively promote sperm storage (38–42).

### Dad*-*Mated Females Show Decoupled Postmating Responses.

Despite initially storing fewer sperm, we confirm previous work in finding that Dad*-*mated females show normal offspring production (31), additionally finding that this holds when females are far more fecund than in previous studies (likely due to the addition of live yeast to the fly food in our experiments, ref. 43) and over both the short- and long-term (linear mixed effects model [LMM], genotype × day, *F*_4, 346_ = 0.305, *P* = 0.875; genotype, *F*_1, 98_ = 0.007, *P* = 0.932; day, *F*_4, 346_ = 49.340, *P* < 0.0001; Fig. 2*D*). We also confirm that Dad*-*mated females show abnormally high receptivity to remating (CPH, *LRT* = 75.158, *P* < 0.0001; Fig. 2*E*), an effect that is absent when flies are kept at low temperatures where Dad overexpression remains inactivated (see *Materials and Methods*; CPH, *LRT* = 0.001, *P* = 0.981; *SI Appendix*, Fig. S3), again supporting the finding that inhibition of BMP signaling in secondary cells reduces male ability to induce refractoriness in their partners. This decoupling in the postmating response is surprising given that both effects are driven by the binding of sex peptide (SP) to a specific receptor expressed in female reproductive tract neurons (44, 45). How these responses are mechanistically uncoupled remains unclear, but it may be that secondary-cell secretions differentially affect interactions between SP and subpopulations of female reproductive tract neurons controlling receptivity (46, 47).

### Females Mated to Dad Males Overretain Sperm in the Seminal Receptacle Despite Normal Offspring Production.

**Fig. 3.**
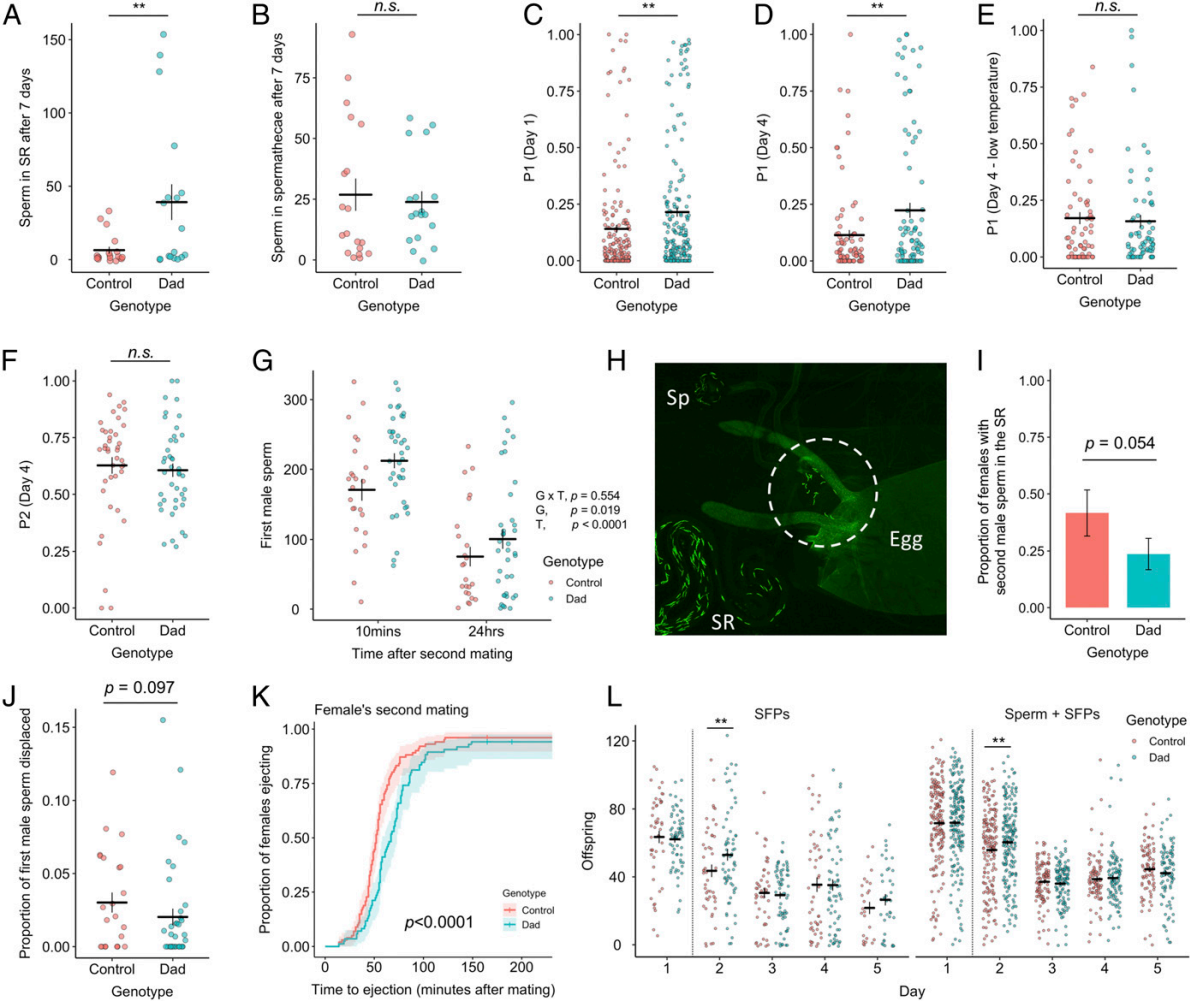
Dad*-*mated females overretain sperm, have higher first male paternity, and handle a second ejaculate differently. (*A*) The number of sperm in the seminal receptacle 7 d after singly mating to a Dad or control male. n_Dad_ = 18, n_control_ = 19. (*B*) As in *A*, but the total across both spermathecae. n_Dad_ = 18, n_control_ = 19. (*C*) First male paternity share when a female first mates to a Dad or control male and then a standardized competitor 24 h later. Offspring collected over the 24 h following remating. n_Dad_ = 190, n_control_ = 173. (*D*) As in *C*, but offspring collected in a 24-h period 4 d after the female remated. n_Dad_ = 92, n_control_ = 81. (*E*) As in *D*, but conducted at 20 °C to block Dad overexpression. n_Dad_ = 69, n_control_ = 67. (*F*) Second male paternity share (P2) when a female first mated to a standardized competitor male and then a Dad or control male 24 h later. Offspring collected in a 24-h period 4 d after remating. n_Dad_ = 43, n_control_ = 41. (*G*) Dad or control sperm across all regions of the female reproductive tract 10 min or 24 h after remating to a standardized competitor. At 10 min: n_Dad_ = 38, n_control_ = 24; at 24 h: n_Dad_ = 38, n_control_ = 24. The *P* values associated with genotype G, timepoint T, and their interaction G x T in predicting sperm numbers are provided. (*H*) A female dissected at 5 h after singly mating to a control male. Released sperm in the uterus are circled. SR, seminal receptacle; Sp, spermathecae. (*I*) Proportion of females where second male sperm has entered into the storage organs 10 min after the start of mating. Females mated to a Dad or control male 24 h previously. n_Dad_ = 38, n_control_ = 24. (*J*) As in *I*, but the proportion of the total first male sperm within the female reproductive tract that is found outside of the storage organs. n_Dad_ = 38, n_control_ = 24. (*K*) Latency to ejaculate ejection after previously Dad- or control-mated females remate with a standardized competitor. n_Dad_ = 85, n_control_ = 101. Confidence interval is 95%. (*L*) Daily offspring production by Dad- and control-mated females that secondarily mate to either a male transferring seminal fluid but no sperm or a normal second ejaculate. The dashed line gives the point at which the female remates. SFPs: n_Dad_ = 66, n_control_ = 48; SFPs + sperm: n_Dad_ = 193, n_control_ = 179. In *A*–*G*, *I*, *J*, and *L*, horizontal bars represent the mean, with vertical bars representing ±1 SE of the mean or proportion. Data are plotted with horizontal “jitter.” **P < 0.01. n.s., not significantly different. n.s. values between 0.05 and 0.1 are provided.

Because Dad*-*mated females store fewer sperm, but produce normal numbers of offspring, we predicted that they would become sperm depleted more rapidly. In contrast, we found significantly more sperm in the primary female sperm storage organ, the seminal receptacle, of Dad*-*mated females 7 d after copulation (LM, *F*_1, 34_ = 12.568, *P* = 0.001; Fig. 3*A*). This effect was independent of the number of offspring produced (LM, genotype × offspring, *F*_1, 33_ = 2.169, *P* = 0.150; offspring, *F*_1, 34_ = 0.429, *P* = 0.517) and did not extend to the spermathecae, where we found no difference in sperm retention (LM, *F*_1, 35_ = 0.005, *P* = 0.947; Fig. 3*B*). This result is only partially consistent with defective activity of SP: females that fail to receive SP are known to show defective release of stored sperm, as are females that receive a form of SP that cannot be cleaved from the sperm surface (48). However, defective SP activity also causes a dramatic reduction in the rate of offspring production (28, 49), which is not exhibited by Dad*-*mated females. Moreover, defects in SP transfer and processing cannot explain the reduction in initial sperm storage in Dad*-*mated females as this process is known to be independent of SP (48). Thus, our data suggest both that 1) Dad*-*mated females show broad decoupling of postmating responses (normal offspring production, but abnormal sperm release and receptivity), and 2) the compromised ejaculate performance of Dad males is wide ranging, affecting both SP-dependent (sperm release, receptivity) and SP-independent (sperm storage) reproductive processes.

### Dad Males Acquire Higher Paternity Shares in Competitive Matings.

*D. melanogaster* females can hold sperm from as many as 6 different males simultaneously (50). However, total female storage capacity is <1,000 sperm, leading to sperm competition between rival males (33). Consequently, males are presumed to be under selection to both displace resident sperm from storage when mating with nonvirgin females (“offensive sperm competition”) and, in turn, to produce sperm that resist displacement by incoming ejaculates (“defensive sperm competition”) (51). To test whether these abilities are mediated by BMP signaling in secondary cells, we first mated a Dad or control male to a virgin female, who then remated 24 h later with a standardized male competitor. Both the females and competitor males carried a recessive *sparkling* (*spa*) eye marker, which allowed us to assign paternity of the resulting offspring (52–55).

We found that Dad males gained significantly higher first-male paternity shares (“P1”) in offspring produced over the first day after female remating (GLM, *F*_1, 360_ = 9.445, *P* = 0.002; Fig. 3*C*). This effect was still present in offspring produced in 24-h periods at day 4 (GLM, *F*_1, 171_ = 11.525, *P* = 0.009; Fig. 3*D*) and day 6 (GLM, *F*_1, 105_ = 7.424, *P* = 0.008) after the female remated. It was also independent of remating latency either overall (GLM, *F*_1, 359_ = 0.264, *P* = 0.608; *SI Appendix*, Fig. S4) or as an interaction with male genotype (GLM, *F*_1, 357_ = 0.329, *P* = 0.567), which suggests that the elevated P1 of Dad males is not an artifact arising through a lack of remating by control-mated females. No P1 differences were detected when flies were kept at low temperatures where Dad overexpression remains inactivated (GLM, day 1, *F*_1, 134_ = 1.717, *P* = 0.192; day 4, *F*_1, 131_ = 1.027, *P* = 0.313; Fig. 3*E*), confirming that the effect is caused by inhibition of BMP signaling in secondary cells. Next, we reversed the mating order, such that Dad or control males mated to a female previously mated to a *spa* male, and found no effect on paternity share (GLM, P2; 24 h, *F*_1, 81_ = 0.246, *P* = 0.621; 4 d, *F*_1, 80_ = 1.814, *P* = 0.182; Fig. 3*F*). Thus, the effect of secondary-cell secretions on sperm competition performance is mating-order specific. More generally, this demonstrates that the mechanisms affecting defensive (P1) and offensive (P2) sperm competition performance can be changed independently of each other.

### Overretention of Dad Sperm Provides a Mechanism for Enhanced Paternity Share.

Under single-mating conditions, Dad*-*mated females retain more sperm 7 d after mating (Fig. 3*A*). Under double-mating conditions, Dad males achieve higher first male paternity shares (Fig. 3 *C* and *D*). Thus, a possible mechanism for the increased paternity share is Dad-mated females having greater numbers of first male sperm in storage at the time of second mating compared to control-mated females. This mechanism would explain why we detect no differences in P2 and would be partially consistent with previous work on failure in secondary cell development, which showed overretention of sperm and improved paternity share (but crucially alongside dramatically reduced offspring production) (28). However, given that Dad*-*mated females initially store fewer sperm (Fig. 2*C*) and display normal productivity (Fig. 2*D*) we predicted a different mechanism: that the elevated paternity share achieved by Dad males acts through enhanced resistance to displacement by a second male ejaculate. To test this, we counted sperm across all regions of the female reproductive tract at 2 timepoints after the start of a female’s second mating: 10 min (approximately halfway through mating) and 24 h. By selecting these timepoints, we were able to ask whether the P1 advantage in Dad-mated females is present from the outset of a female’s second mating or whether it develops over the course of second male sperm entering into storage.

Overall, we found significantly higher quantities of first male sperm throughout the female reproductive tract (in storage or displaced into the uterus) in Dad-mated females (LM, *F*_1, 120_ = 5.616, *P* = 0.019; Fig. 3*G*). This effect was independent of the timepoint after mating (LM, genotype × timepoint, *F*_1, 119_ = 0.351, *P* = 0.554; Fig. 3*G*), but contrary to our prediction, there was a trend for the degree of difference between Dad and control sperm number to be diminished 24 h after remating. Thus, the P1 sperm advantage in Dad-mated females appears to be present at the start of a female’s second mating and, if anything, remating appears to weaken, not reinforce the sperm advantage of the Dad male. This also means that despite Dad-mated females initially storing reduced quantities of sperm (Fig. 2*C*), they hold more in storage relative to control-mated females by the time of their second mating (Fig. 3*G*). Greater retention of sperm is a known consequence of SP dysregulation, but in these cases it is partly explained by females using fewer sperm because they produce fewer offspring (28, 48). Why, then, does reduced sperm release in Dad*-*mated females not translate into reduced offspring output (Fig. 2*D*)? The most parsimonious explanation is that Dad*-*mated females achieve the same number of fertilizations as control-mated females, but release fewer sperm per fertilization. Previous estimates suggest that females release 1 to 5 sperm per fertilization, but are able to modulate the efficiency of sperm use in response to variation in environmental quality (56). While sperm use is challenging to measure directly, on the rare occasions where we found eggs in the uterus of dissected females we did find instances where large numbers of sperm (up to 17 sperm) were associated with an egg (Fig. 3*H*), suggesting that sperm use may be more inefficient than previously suggested. This inefficiency may be particularly pronounced when the storage organs are largely full, as would be the case so soon after mating (5 h). Despite appearing wasteful, profligacy in sperm release may be adaptive if it encourages further competition between sperm of varying quality, with consequences for offspring fitness (57–59). However, profligacy also serves to more rapidly deplete the sperm storage organs, giving an increased advantage to the second mating male. Thus, the rate of female sperm use is likely to be a crucial mediator of the intensity of postcopulatory sexual selection.

### Altered Dynamics of Second Male Ejaculates in Dad-Mated Females.

Dad-mated females treat potential sexual partners differently by showing higher receptivity to remating. We therefore sought to test whether they treat second male sperm differently. We first looked at the rate at which second male sperm are stored. It is already known that if a male fails to transfer Acp36DE, both his sperm and those transferred by the next male show compromised storage, despite the second male presumably transferring Acp36DE himself (10). Dissecting females 10 min after starting a second mating, we found a nonsignificant trend for slowed entry of second male sperm in previously Dad-mated females (GLM, *F*_1, 59_ = 3.718, *P* = 0.054; Fig. 3*I*) and reduced displacement of first male sperm at this timepoint (first male sperm in the uterus/total first male sperm across all regions of the reproductive tract; GLM, *F*_1, 61_ = 2.836, *P* = 0.097; Fig. 3*J*).

We next tested for differences in the timing of female ejection. The length of time a female retains a second male ejaculate after remating influences the outcome of sperm competition: the longer it takes a female to eject, the greater the opportunity for second male sperm to enter into storage and displace resident sperm (60). We therefore predicted that Dad-mated females would eject sperm earlier, thereby terminating the displacement of first male sperm, and promoting the paternity share advantage experienced by Dad males (Fig. 3*C*). Contrary to expectation, Dad-mated females were significantly slower to eject after their second mating (CPH, *LRT* = 17.981, *P* < 0.0001; Fig. 3*K*), despite showing no change in ejection timing when initially receiving a Dad male’s ejaculate (*SI Appendix*, Fig. S2). This should weaken the advantage experienced by Dad males that arises through overretention of sperm by their female partners. Indeed, this weakening could explain the slight decrease in the degree of difference between Dad and control sperm number in the 24 h after remating relative to 10 min after remating (Fig. 3*G*). Ultimately, this result suggests that female treatment of a second ejaculate is influenced by features of the first male’s ejaculate.

Finally, we tested whether offspring production after a second mating differs depending on whether a female first mated with a Dad male or a control. As second males we used either males transferring both sperm and seminal fluid or spermless *son-of-Tudor* males that transfer seminal fluid but no sperm. This allowed us to identify the relative importance of second male sperm and seminal fluid in driving any detected effects. We found a significant interaction between day since mating and first male genotype on daily offspring production (LMM, *F*_4, 1432_ = 2.740, *P* = 0.027; Fig. 3*L*). This appears to be driven by a short-term increase in offspring production by Dad-mated females exclusively in the 24 h following remating (*t* ratio *=* 2.663, *P* = 0.008). This effect was independent of whether the female received second male sperm (LMM, first male × second male × day, *F*_4, 1398_ = 0.577, *P* = 0.679; first male × second male, *F*_1, 400_ = 0.096, *P* = 0.757), demonstrating that it is specifically attributable to the second male’s seminal fluid. A potential mechanism for this short-term boost in offspring production in Dad-mated females is second males transferring larger quantities of fecundity-stimulating SFPs when mating with Dad*-*mated females compared to those females previously mated to controls. There is good precedent for this: males strategically decrease their transfer of the short-term acting, fecundity-stimulating SFP ovulin when they detect that they are mating with a mated female (61). Given the high receptivity of Dad-mated females, second males may perceive them as virgin-like and transfer higher quantities of SFPs such as ovulin, though this remains to be tested. However, male detection of female mating status is at least partly achieved through changes in the female pheromonal profile after mating (62). Thus, this mechanism would require that Dad-mated females show a divergent postmating pheromonal profile or for males to integrate additional non-olfactory cues when assessing female mating status.

### The SFP Proteome Is Remodelled in Dad Males.

**Fig. 4.**
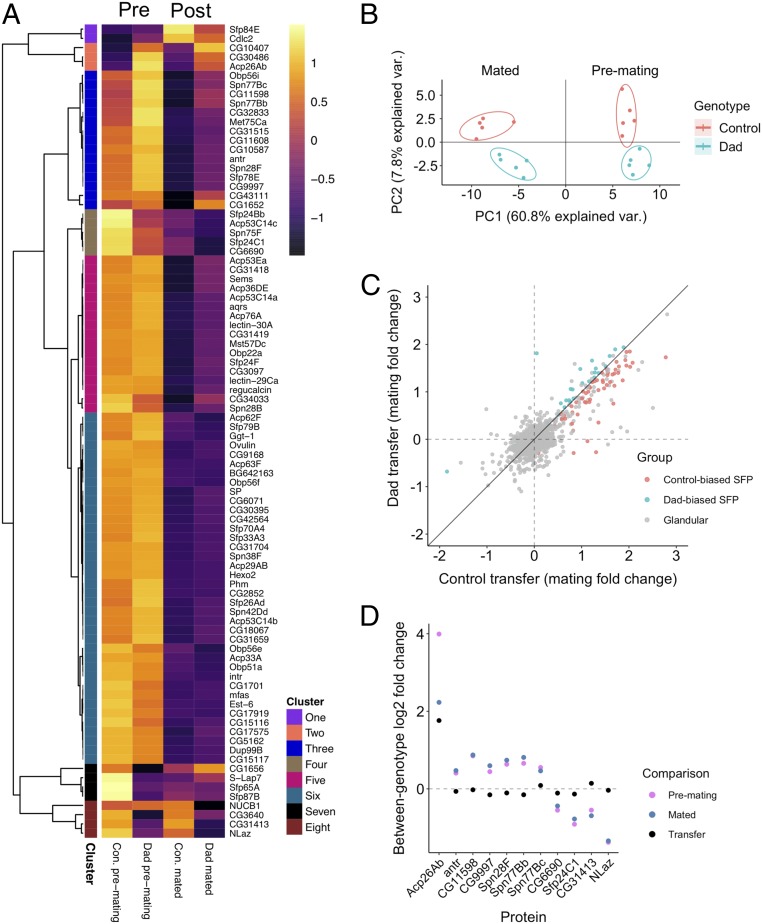
Quantitative proteomics reveals remodeling of the SFP proteome in Dad males. (*A*) A heatmap showing the abundance patterns of SFPs. Columns 1 and 2: males dissected prior to mating; columns 3 and 4: males dissected 25 min after mating. Columns 1 and 3: control males; columns 2 and 4: Dad males. Row annotations highlight membership of higher-order clusters based on a Pearson correlation distance metric. (*B*) Output of a PCA conducted on abundances of the 88 detected SFPs. Points colored according to male genotype. Mated glands are on the *Left*, premating glands on the *Right* of *x* = 0 line. Ellipses denote 80% normal probability. (*C*) Correlation between Dad and control pre- vs. postmating fold changes (degree of transfer) for each SFP. Red gives SFPs transferred in greater quantities by control males, blue gives SFPs transferred in greater quantities by Dad males. Gray denotes glandular proteins (i.e., non-SFPs). (*D*) Log_2_ fold changes for 3 different between-genotype comparisons for each of 11 SFPs identified as showing a significant abundance change in response to BMP signaling suppression. Comparisons: premating (pink), postmating (blue), and transfer to females (black). Positive values indicate greater abundance in Dads.

The phenotypic effects we find in Dad-mated females are likely to arise through changes to the production, transfer, and protein composition of seminal fluid, particularly given that BMP signaling promotes secondary-cell secretion (27, 32). This change may operate exclusively through secondary cells or, if there is cross-talk between cell types, also via their influence on main cells. To this end, we performed label-free quantitative proteomics on the accessory glands of Dad and control males dissected either before or immediately after mating. This pre- and postmating approach has previously been shown to provide a deep analysis of the seminal proteome that is sensitive to low abundance proteins and which can expose patterns of differential SFP production, depletion, and transfer (19, 54, 63). We detected 1,194 proteins on the basis of at least 2 unique peptides (as in refs. 19, 54, 63, and 64), of which 88 are SFPs known to be transferred to females (*Materials and Methods*). A principal component analysis (PCA) conducted on these 88 SFPs showed full separation of samples in relation to both genotype and mating status (Fig. 4*B*). Analysis of the extracted scores showed that PC1, which described the majority of variance (60.8%), was associated with the interaction between mating and genotype (*SI Appendix*, Table S1). PC2 was significantly described by male genotype and captures an axis of variation (7.8%) associated with divergent responses among SFPs in the extent to which their abundance was affected by secondary cell disruption. Thus, as expected, inhibition of BMP signaling in secondary cells changes the SFP composition of the accessory glands.

### Split Responses of the Seminal Proteome to Suppression of Secondary Cell BMP Signaling.

To test for patterns among SFPs in their response to BMP signaling suppression in secondary cells, we undertook a hierarchical clustering analysis across genotypes and mating treatments (Fig. 4*A*). Responses of SFPs to genotype appear variable with multiple higher-order clusters identified. The changes did not suggest a complete loss of any SFPs in Dad males. Instead, we find evidence of quantitative changes in the abundance of some SFPs. Indeed, we find that a majority of SFPs are transferred in smaller quantities in Dad males compared to controls (67% of SFPs show a smaller pre- vs. post-mating change in Dad males; 2-tailed binomial test, *P* = 0.002; Fig. 4*C*). Following false detection rate (FDR) correction, we failed to identify any SFPs showing the significant mating × genotype interaction that would indicate high-confidence differences in transfer. This may in part be due to low power (5 samples per treatment combination), but it could also be due to any differences in transfer being relatively small, which seems to be the case for most SFPs (Fig. 4*C*). However, we found that 11 of the 88 SFPs show a significant response to genotype (Fig. 4*D* and *SI Appendix*, Table S2 and Fig. S5). This list did not include SP or Acp36DE, 2 candidate proteins that could be influencing the receptivity (Fig. 2*E*) and sperm storage (Fig. 2*C*) phenotypes, respectively, that we detect in Dad-mated females. A further 26 differentially abundant glandular proteins (i.e., non-SFPs) are given in *SI Appendix*, Table S3. Thus, while SFPs make up just 7.4% of the proteins we detect (88/1,194), they make up 29.7% (11/37) of the proteins showing a significant difference in abundance in Dad males, suggesting a disproportionate effect of BMP signaling suppression on the seminal fluid proteome.

Seven of the 11 differentially abundant SFPs showed higher abundance in Dad glands (Acp26Ab, antr, CG11598, CG9997, Spn28F, Spn77Bb, and Spn77Bc), 4 showed higher abundance in control glands (CG6690, Sfp24C1, CG31413, and NLaz). CG9997 is thought to be specifically expressed in secondary cells, but we did not find significant differences in abundance in other SFPs thought to be produced in the secondary cells, such as CG1652, CG1656, and CG17575 (28). Therefore, suppression of BMP signaling does not appear to block production of these secondary cell proteins, and its effects on their abundance seem to be selective.

Acp26Ab stands out from the other differentially abundant SFPs in the scale of its expression differences: 16× more abundant in pre-mating Dad glands and 8× more abundant in post-mating Dad glands. This suggests that Dad males increase the transfer of this SFP. Consistent with this, Acp26Ab had the lowest FDR-corrected genotype × mating *P* value of the 1,194 proteins we tested (LM, *P* = 0.059). Interestingly, previous work has shown that Acp26Ab is present in both main and secondary cells within the first day of eclosion, but after 5 d is only present within the dense core granules of secondary cells (65), a pattern that suggests Acp26Ab is produced by main cells and trafficked to secondary cells. Therefore, it may be that suppression of BMP signaling in secondary cells disrupts this process of intercellular transport leading to overproduction of Acp26Ab by main cells.

Like Acp26Ab, CG11598 has also been shown to be present in both main and secondary cells. In a previous transcriptomic study, manipulation of secondary cell development led to a large down-regulation of *CG11598* expression, the magnitude of which was suggested to only be accountable for by changes in main-cell activity (21). Surprisingly, we found that the abundance of CG11598 changed in the opposite direction, being significantly more abundant following suppression of secondary cell BMP signaling. Collectively, the changes we detect in Acp26Ab and CG11598 suggest a role for the secondary cells in mediating the activity of main cells, perhaps via cell–cell signaling. Future work should seek to identify whether these changes to main cells adaptively buffer against more extreme phenotypic consequences of defective secondary-cell activity, such as compromised fertility, or whether they themselves contribute to the various phenotypic abnormalities we detect in Dad-mated females.

In 8 of 11 of these proteins, the between-genotype fold change became more Dad biased after mating (blue dot above pink dot, Fig. 4*D*). Indeed, looking across all 88 SFPs, we find that the majority of SFPs are at higher abundance in Dad glands prior to mating (65%, 57/88; 2-tailed binomial test, *P* = 0.007) with the number increasing after mating (73%, 64/88; 2-tailed binomial test, *P* < 0.0001). We offer 2 explanations for why the majority of SFPs are initially at higher abundance in Dad males. Firstly, Dad males may overproduce SFPs, perhaps due to disruption to main-cell/secondary-cell signaling. Secondly, if males suffer even slightly reduced SFP transfer in each mating then they may accumulate overretained SFPs following the previous day’s triple matings, which we provided to clear the glands of products produced prior to expressing Dad (*Materials and Methods* and as in refs. 27 and 31). In either case, the differences in transfer for the significantly differentially abundant SFPs are surprisingly small, given the clear between-genotype differences in their abundance within the gland (Fig. 4*D*). This suggests that there may be mechanisms that regulate the quantity of accessory gland secretion that is transferred to females independently of both the quantity within the gland and secondary-cell activity.

## Conclusions

We conclude that BMP signaling in adult secondary cells is a major mediator of manifold reproductive processes. These findings have broad implications for our understanding of how ejaculates evolve. Firstly, ejaculate evolution appears to be constrained. Although normal secondary-cell activity inhibits male defensive sperm competition performance, it is required to reduce female receptivity to remating. Given that the latter ability is the wild-type condition, it seems likely that the benefits that loss of secondary-cell secretion brings to paternity share are outweighed by the benefits of suppressing female receptivity to remating. However, the question remains as to why males apparently aren’t able to simultaneously maximize performance in both. Such intraejaculate trade-offs in function may represent an underappreciated constraining force on ejaculate evolution. Secondly, our data demonstrate that the composition and function of the ejaculate depends on the integrated activity of the 2 constituent cell types of the accessory glands. Thus, evolutionary changes to the cellular architecture of seminal-fluid-producing tissues should have knockon consequences for ejaculate composition and function. Interestingly, secondary cell number is variable between *Drosophila* species—they have even been lost entirely in *Drosophila grimshawi* (15). In light of our results, we would predict covariance between accessory gland cellular architecture and variable aspects of mating biology, such as mating rate and sperm competition intensity, across the *Drosophila* phylogeny. Given that we find an element of modularity in ejaculate design, with normal offspring production being exclusively driven by main-cell activity in adults, it may be that some reproductive functions are insulated from changes in a given part of the male reproductive system. Ultimately, by taking an evo-devo approach to male reproductive tissues we may begin to understand how ejaculate function and composition evolve.

## Materials and Methods

### Fly Stocks and Husbandry.

Males with disrupted secondary-cell secretion were generated by crossing esg^ts^ F/O flies (genotype: *w; esg-GAL4 tub-GAL80*^*ts*^ *UAS-FLP/CyO; UAS-GFP*_*nls*_ *actin > FRT > CD2 > FRT > GAL4/TM6*) to *w*^*1118*^ flies into which a *UAS-Dad* transgene had been backcrossed (“Dad” males) (27, 31). For controls, we crossed esg^ts^ F/O to flies from a *w*^*1118*^ background (“control” males). The *esg-GAL4* system incorporates a temperature-sensitive GAL80, which inhibits GAL4 and suppresses the activation of Dad overexpression below 28.5 °C (see ref. 31). Where sperm counts were undertaken, we backcrossed the *GFP-ProtB* construct, which labels the heads of sperm (33), into our Dad and *w*^*1118*^ lines for 6 generations. All females were from a Dahomey wild-type background into which the *spa*^*pol*^ recessive eye marker had previously been backcrossed for 4 generations. All competitor males were of this same genotype or, where sperm counts were conducted, this genotype carrying a *RFP-ProtB* construct (33).

All flies were reared at standardized larval densities of ∼200 in 250-mL bottles containing 50 mL of Lewis medium (as in ref. 66). Larvae were left to develop at a nonpermissive temperature (i.e., at which GAL4 remained inactive) of 20 °C on a 12:12 L:D cycle. Upon eclosion, we collected males under ice anesthesia and separated them into groups of 8 to 12 in 36 mL Lewis medium-containing plastic vials, supplemented with ad libitum yeast granules. To activate the overexpression of Dad, we immediately moved these vials to 30 °C where they remained for the full duration of experiments. Thus, Dad and control males were placed at the permissive temperature, whereupon the *GAL4* system is activated, within hours of eclosion, and held there constantly. Although this temperature can negatively impact sperm in some temperature-intolerant strains of *D. melanogaster* (67), the high sperm storage and fertility we measured—comparable or exceeding previous studies conducted at lower temperatures (28, 34)—suggests that our strains are high-temperature tolerant, and that any effects must be relatively small. Crucially, control flies were treated identically, meaning that any temperature effects do not confound the results. To verify that phenotypes were specifically attributable to Dad overexpression, we repeated some experiments at a nonpermissive temperature of 20 °C. In these experiments, flies were maintained at 20 °C after eclosion (i.e., the same as the rearing temperature) where they remained for the full duration of experiments. The day before using Dad or control males in experimental matings, each male was successively mated to 3 virgin females. We used this to deplete, as much as possible, the accessory gland lumen of any secondary cell products produced before expression of the *Dad* transgene was activated. We delivered a single female at a time, removing the female after mating. Following the end of the third mating, we moved the male to a fresh, yeast-supplemented vial.

The rearing, collection, and grouping of flies from all lines that did not carry temperature-sensitive transgenes (i.e., females and competitor males) were performed following the methods outlined above. However, in these cases rearing was conducted at 25 °C with us moving flies to 20 °C or 30 °C (depending on whether we were using a permissive or nonpermissive temperature for the GAL4 system) the evening before use in experiments. We reared all flies and performed all experiments in controlled-temperature rooms on 12:12 light:dark cycles. All flies were between 3 and 5 d old at the time of first experimental mating.

### Sperm Count Experiments.

We conducted the initial sperm transfer experiment in 2 blocks. Females were frozen at 25 min or 5 h after the start of mating. We conducted the sperm retention experiment in 1 block. Here, females were frozen 7 d after mating. We conducted the competitive sperm dynamics experiment in 2 blocks. Here, females were frozen at 10 min or 24 h after second mating. Females in all experiments were randomly assigned a freezing timepoint prior to mating. Offspring were collected and counted between mating and freezing where appropriate. Females were flash frozen in liquid nitrogen and stored at −80 °C until dissection, which we performed under light microscope in PBS. We retained the female reproductive tract from the vulva through to the common oviduct, sealed the slides using (Fixogum, Marabu), and stored slides at 5 °C. We imaged the slides using a Zeiss 880 confocal microscope and processed the images by taking an average intensity Z projection in the Fiji distribution of ImageJ (68) to condense Z stacks into a single image for easier counting. We manually counted sperm using the multipoint tool in Fiji. We performed all dissections and sperm counts blind to treatment. We omitted any samples that showed no GFP sperm due to the possibility of heterozygosity for the *GFP-ProtB* chromosome in our stock populations.

### Sperm Competition Outcome and Postmating Response Assays.

For P1 defensive sperm competition assays, we aspirated single Dad or control males into yeasted vials containing an individual virgin *spa*^*pol*^ female. We monitored all matings, recording the time males were introduced, mating began, and when mating finished. From these data we calculated the duration of and latency to mating. After mating, we disposed of the males and left the females to oviposit. The following morning, we individually aspirated mated females into a yeasted vial containing a pair of *spa*^*pol*^ males, grouped under ice anesthesia the previous day. Again, we monitored all matings and recorded duration and latency. We introduced females in the order they had finished mating the previous day. Previous work has shown that Dad*-*mated females remain highly receptive to remating (31), so we staggered the introduction of Dad*-*mated females to minimize any systematic difference between treatments in intermating interval. Following the end of mating, we discarded the 2 males and moved the females to 25 °C, transferring them into a fresh, yeasted vial every 24 h. We allowed the resulting progeny to develop, freezing the vials after the adults eclosed. We then counted offspring and scored their eye phenotype in order to assign paternity. By adopting this same approach but reversing the mating order, we tested for an association with offensive sperm competition performance (P2). We performed 3 blocks of a repeat of the P1 experiment conducted entirely at a nonpermissive temperature of 20 °C. We obtained P1 data across 6 experimental blocks at 30 °C. In each of these, we collected offspring for at least 24 h after the female’s second mating. In 1 replicate, we collected offspring for 6 d to test for the persistence of any detected differences. Within 4 of these replicates, we added 2 further treatments: while some Dad- and control-mated females secondarily mated to *spa*^*pol*^ males, others received either no second mating or a spermless, *son-of-Tudor* mating. We randomly assigned females to each of these mating treatments the day before use in experiments. In these 4 replicates, we collected offspring over 4 d after second mating to gain additional information relating to short- and longer-term patterns of offspring production.

### Female Ejection Assays.

We followed the P1 experimental setup outlined in the preceding section, but moved females to 3D-printed, black plastic chambers immediately after a first or second mating. These chambers, of printing resolution 0.2 mm, were cuboids of 34 mm × 33 mm × 9 mm with a half-sphere concavity of dimensions 20 mm × 20 mm × 7 mm. A .stl file of this design is included alongside our deposited datasets on the Oxford Research Archive for use by other researchers. We used a glass coverslip to cover the concavity once a female had been introduced. We checked each chamber for the presence of an ejected sperm mass every 10 min under a light microscope (as in ref. 60). We ran this experiment 4 times: twice for each of the females’ first (Dad or control) and second (*spa*^*pol*^) mating.

### Proteomics Experiment.

Dad and control males were reared, collected, temperature shifted, and triple mated exactly as described for the previous phenotyping experiments. On the day of the experiment, we randomly assigned males a mating treatment (“premating” or “mated”) and paired them within a genotype. We aspirated the mated-treatment male within each pair into a yeasted vial containing an individually isolated 4/5-d-old virgin female. At this same point, the premating male from the pair was introduced to an empty, yeasted vial. We flash froze mated males in liquid nitrogen 25 min after the start of mating, freezing their premating partner at the same time. This paired-freezing approach ensures that the distribution of freezing times is equivalent between mated and premating males (as in ref. 63). Frozen males were stored at −80 °C until dissection.

For each sample, we pooled 20 pairs of accessory glands, which we dissected under a light microscope on ice in a drop of ice-cold PBS. We took care to remove the seminal vesicles and testes and severed the glands from the distal end of the ejaculatory duct. Dissected glands were then transferred to an Eppendorf tube containing 25 µL of PBS, which we stored at −80 °C. In total, we had 20 samples: 5 for each of the 4 treatment permutations (mated, Dad; premating, Dad; mated, control; and premating, control). We ran this experiment 5 times in order to produce 5 independent biological replicates. Our quantitative proteomics analysis was conducted in accordance with the gel-aided sample preparation (GASP) protocol outlined in detail elsewhere (19, 69). Details of this method, the LC-MS/MS platform, and the data processing and normalization are given in *SI Appendix*. The mass spectrometry proteomics data were deposited in the ProteomeXchange Consortium via the PRIDE (70) partner repository with the dataset identifier PXD015253.

### Statistical Analysis.

We conducted all analyses with R statistical software (version 3.5.1) (71) in RStudio (version 1.1.456) (72). We assessed the significance of variables in linear and generalized linear models by dropping individual terms from the full model using the “drop1” function, which compares models based on the Akaike information criterion (AIC). Where the interaction term was nonsignificant we refitted the model without it. We determined model fit by visual inspection of diagnostic plots (73). Where multiple measurements were taken from the same female, as in analyses of day-by-day female offspring production, we used linear mixed effects models that accounted for female identity as a random effect. In our day-by-day analysis of female offspring production, our starting model contained a 3-way interaction (male 1 × male 2 × day) along with 2 random effects (block and female ID). We used a stepwise algorithm (“step” function) to identify the best model by AIC. Associated *P* values were generated using Satterthwaite’s method (74). To analyze latency to mating and ejection, we ran Cox proportional hazard models using the *survival* package (75, 76) and graphed the results using “ggsurvplot” in the *survminer* package (77). We analyzed proportional data, relevant for paternity shares (P1 and P2) and some sperm count data, using generalized linear models. In all cases, we used a quasibinomial extension to account for the overdispersion we detected, which is commonly encountered in sperm competition data (e.g., refs. 78–80) at least in part due to the presence of excess zero values. When analyzing the number of sperm retained in the seminal receptacle after 7 d, we used a quasipoisson distribution to correct for overdispersion. We limited all analyses to matings lasting longer than 7 min, which gave rise to fertile offspring to exclude disturbed or pseudomatings (81). In our analysis of first male sperm retention after a second mating, we winsorized 1 extreme significant outlier (as determined by 2-tailed Grubbs’ test) found to exert disproportionate leverage in our models (82).

Our assessment of whether a protein was a SFP was based on a reference list provided by Mariana Wolfner (Cornell University, Ithaca, NY) and Geoff Findlay (College of the Holy Cross, Worcester, MA) and updated to include the high confidence SFPs from Sepil et al. (19). We also included Intrepid (intr), despite it not having been conclusively shown to be transferred to females, as we find it at significantly lower abundance in mated glands and because it is known to function in the sex peptide network (16). All analyses were performed on log_2_ transformed values to standardize the variance across the dynamic range of protein abundances. Fold changes were calculated using per-treatment means (taken across the 5 replicates). Our hierarchical clustering analysis was conducted on the mean per-SFP abundance taken across the 5 replicates for each treatment permutation and used a Pearson correlation distance metric. We plotted the results using the *pheatmap* package (83). We conducted a PCA on SFPs using the “prncomp” function in *stats*. Each protein’s values were scaled to have unit variance and shifted to be zero centered. We ran linear models on the PC scores to test for associations between PCs and our variables. For our differential abundance analysis, we iterated a linear model over all detected proteins across the 20 samples, including genotype, replicate, and mating status as factors. We used a tail-based false discovery rate correction from the *fdrtool* package (84).

## Supplementary Material

Supplementary File
