# Supplementary material for: BMP signaling inhibition in Drosophila secondary cells remodels the seminal proteome and self and rival ejaculate functions
Source: Proc Natl Acad Sci U S A. 2019 Nov 18;116(49):24719–28. doi: 10.1073/pnas.1914491116 (PMC6900634; doi:10.1073/pnas.1914491116)
Supplement: Supplementary File [file pnas.1914491116.sapp.pdf]

PNAS
www.pnas.org

Supplementary Information for

BMP-signalling inhibition in *Drosophila* secondary cells remodels the seminal proteome, and self and rival ejaculate functions

Ben R. Hopkins, Irem Sepil, Sarah Bonham, Thomas Miller, Philip D. Charles, Roman Fischer, Benedikt M. Kessler, Clive Wilson, and Stuart Wigby

Corresponding author:
Ben R. Hopkins

Email: brhopkins92@gmail.com

This PDF file includes:

Supplementary information text
References for SI
Figs. S1 to S5
Table S1 to S3

www.pnas.org/cgi/doi/10.1073/pnas.1914491116

Supplementary Information Text
Materials and Methods

Proteomics sample preparation

We prepared our samples for proteomic analysis in line with the previously published GASP protocol (1,2). The glandular tissue was first macerated using a clean pestle for a timed interval of one minute. To lyse the cells, we added 25µl of Pierce RIPA (Radioimmunoprecipitation Assay) Buffer, which we dripped over the pestle to flush residual tissue back into the sample. Next, we incubated the lysate with 50mM of the reducing agent DTT (Dithiothreitol) for approximately 10 to 20 minutes. To this, we added at room temperature an equal volume of 40% acrylamide/Bis solution (37.5:1 National Diagnostics) to facilitate cysteine alkylation to propionamide. Next, we added 5µl of 10% APS (ammonium persulphate) and an equivalent quantity of TEMED (tetramethylethylenediamine) to trigger the polymerization of acrylamide and form a gel plug. This plug was subsequently shredded via centrifugation through a membrane-less Spin-X filter insert (CLS9301, Sigma/Corning). Gel-fragments from this process were fixed in 40% ethanol/5% acetic acid before washing with a solution of 50mM ammonium bicarbonate, 1.5M Urea, and 0.5M Thiourea, which was then removed with acetonitrile. 250µl of dilute trypsin (Promega) was next added and the solution left at 37°C overnight to promote digestion of the immobilised peptides. The resulting peptides were extracted via two repeated ACN (acetonitrile) replacements, dried, desalted in Sola SPE columns (Thermo), and then suspended in 0.1% FA (formic acid), 2% ACN before LC-MS/MS (liquid chromatography-mass spectrometry/mass spectrometry) analysis.

LC-MS/MS. For peptide analysis, we used a LC-MS/MS platform composed of a Dionex Ultimate 3000 and a Q-Exactive mass spectrometer (Thermo). Peptide loading took place in a solution of 0.1% TFA (trifluoroacetic acid) in 2% ACN on a trap column (PepMAP C18, 300µm x 5m, 5µm particle, Thermo). For separation, we used an easy spray column (PepMAP C18, 75µm x 500m, 2µm particle, Thermo) with a gradient 2% ACN to 35% ACN in 0.1% FA in 5% DMSO (dimethyl sulphoxide). For MS spectra collection, we used a resolution of 70,000 in profile mode on the Q-Exactive (ion target = $3x10^6$). We selected the top 15 most intense features selected for subsequent MS/MS analysis (resolution of 17,500). The following parameters were set: dynamic exclusion = 27 seconds; AGC target = $1x10^5$; isolation width = 1.6m/z; and maximum acquisition time = 100ms.

MS data processing. The following MS data processing pipeline has previously been outlined in Sepil et al. (2). We imported the RAW data into Progenesis QIP (version 4.1.6675.48614), exporting spectra as MGF files using the 200 most intense peaks without deconvolution for searching. Peptide identification used the D. melanogaster UniProt reference proteome as a search target, with database retrieval conducted on 27/09/2017 (23306 sequences) in Mascot 2.5.1. Search parameters were set as follows: Oxidation (M),

Propionamide (K), and Deamidation (N,Q) as variable modifications; Propionamide (C) as a fixed modification; one missed cleavage site; 0.05 Da fragment mass accuracy; 10ppm precursor accuracy. Prior to importing the search results into Progenesis for quantification via the Top3 method, we applied a peptide-level 1% FDR alongside a further Mascot ion cut-off of 20. The resulting protein abundance data was subsequently normalised using the internal Progenesis algorithm to a set of housekeeping proteins.

Supplementary References

1. Fischer R, Kessler BM (2015) Gel-aided sample preparation (GASP)-A simplified method for gel-assisted proteomic sample generation from protein extracts and intact cells. Proteomics 15(7):1224–1229.
2. Sepil I, et al. (2019) Quantitative proteomics identification of seminal fluid proteins in male Drosophila melanogaster. Mol Cell Proteomics 18(Supplement 1):S46–S58.

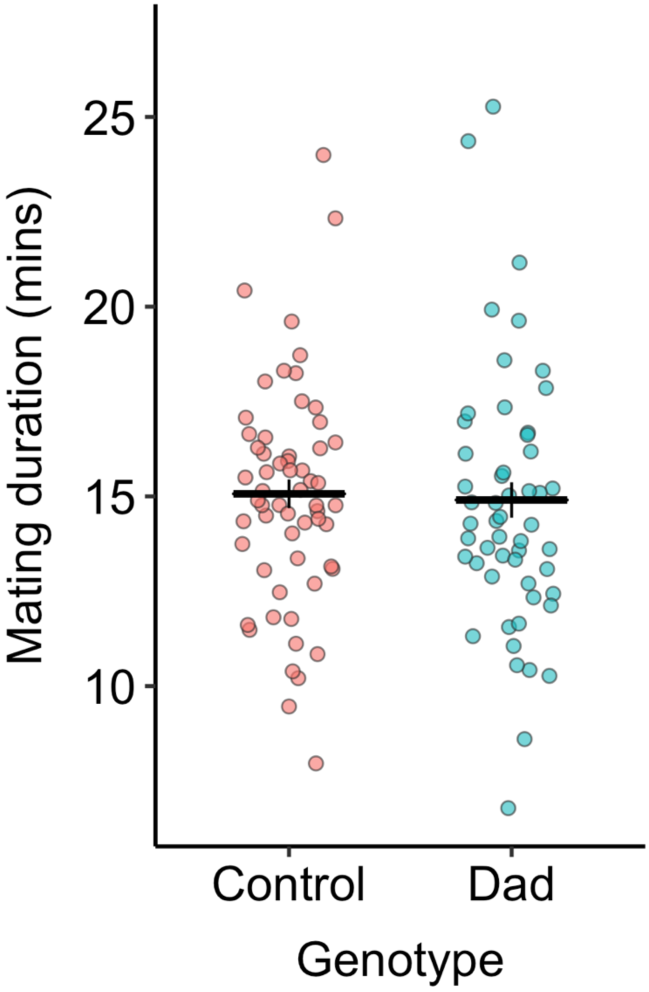

Figure S1. No significant difference in the duration of mating between Dad and control males. n_{Dad}=53, $n_{Control}$=59. Horizontal bars represent the mean, with vertical bars representing ± 1 SE. Data are plotted with horizontal 'jitter'. Data pooled from two experimental blocks. p=0.787.

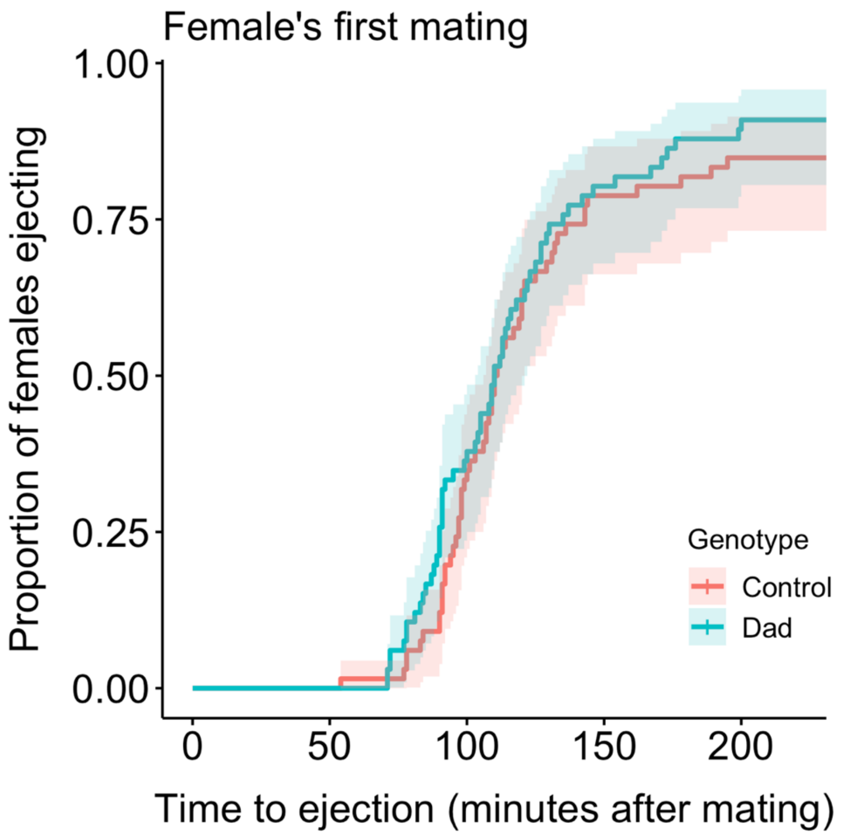

Figure S2. No significant difference in the timing to ejaculate ejection after a female singly mates with either a Dad or control male. n_{Dad}=66, $n_{Control}$=66. Confidence intervals are at 95%. Data pooled from two experimental blocks. p=0.345.

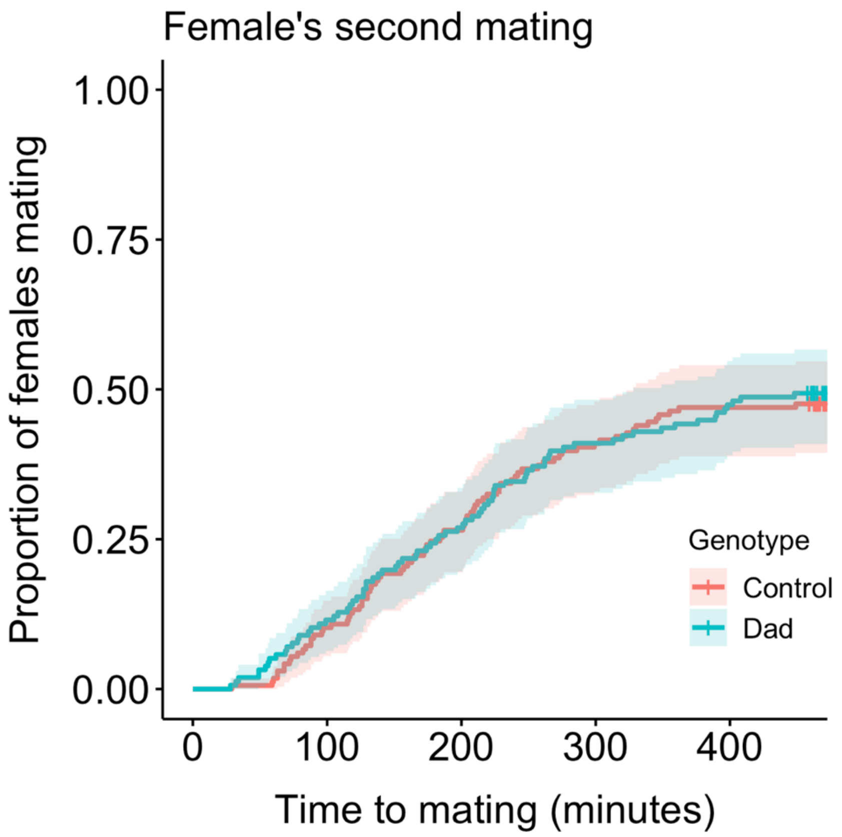

Figure S3. No difference in the latency to remating after previously Dad- or control-mated females are provided with a second mating opportunity 24 hours later. Here, both Dad and control males were reared at temperature of 20°C where Dad is not expressed. n_{Dad}=156, $n_{Control}$=166. Confidence intervals drawn at 95%. Data pooled from three experimental blocks. p=0.981.

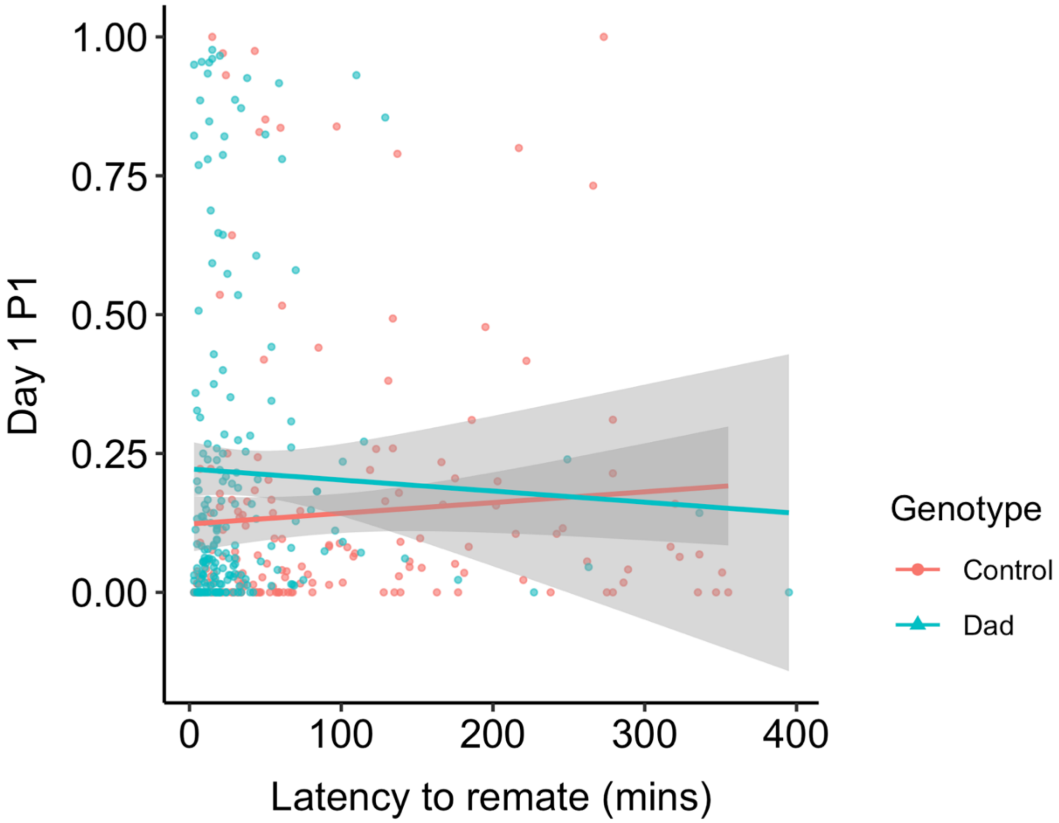

Figure S4. A regression of first male paternity share against latency to remating by Dad- (blue) and control- (red) mated females secondarily mated to a standardised competitor. n_{Dad}=190, $n_{control}$=173, pooled across 6 blocks. Confidence intervals drawn at the 95% level. Significant effect of genotype (*p*=0.001), but not of latency (*p*=0.608) nor the interaction between them (*p*=0.567).

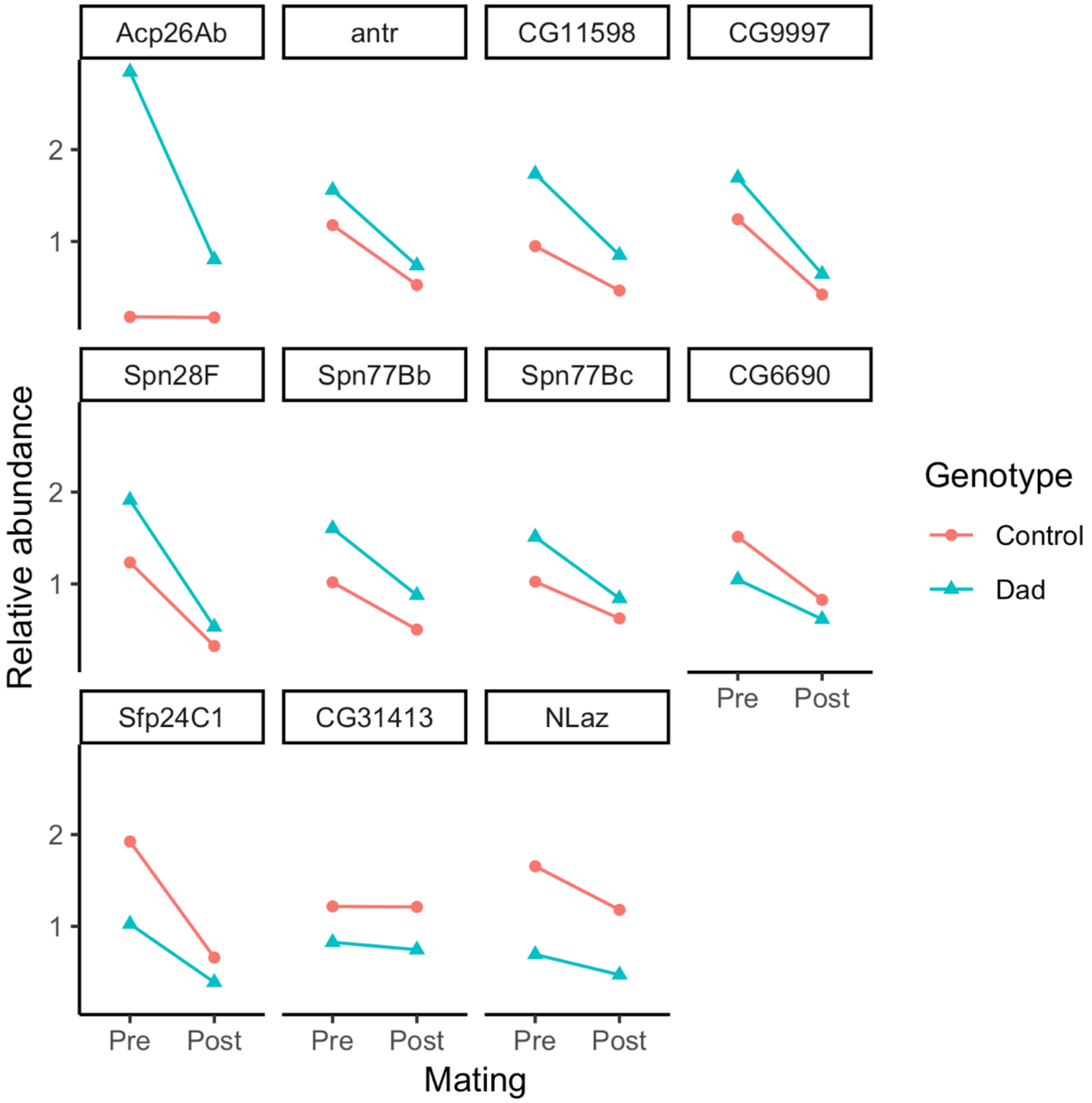

Figure S5. Abundance profiles of SFPs identified as differentially-abundant in relation to genotype (FDR p<0.05). Each point represents an average across the 5 replicates in relation to genotype and mating status. The abundance values are relativized by means-centring and averaging across replicates.

Table S1. Summary statistics from a PCA conducted on detected SFPs. (A) The variance, eigenvalue, and loadings associated with the first four principal components (PCs). (B) The output from linear models fitted to each of the first three PCs, using the measured variables of genotype (Dad or control), mating status (mated or pre-mating), and replicate (5 in total). Significant associations at the $p<0.05$ levels are given in red.

A

	PC1	*PC2*	*PC3*	*PC4*
Variance explained (%)	60.76	7.77	6.15	4.35
Eigenvalue	53.47	6.84	5.41	3.83
No. positive loadings	82	42	47	44
No. negative loadings	6	46	41	44

B

PC1 *Effect*	*Df*	*Sum of sq*	*RSS*	*F*	*P*
*Genotype*Mating*	1	3.6962	11.3870	5.7672	0.0334
Genotype	1	18.56	29.95	21.190	0.0004
Mating	1	971.50	982.88	1109.1	<0.0001
Replicate	4	14.52	25.90	4.1433	0.0222

PC2 *Effect*	*Df*	*Sum of sq*	*RSS*	*F*	*P*
*Genotype*Mating*	1	1.1368	9.4912	1.6329	0.2255
Genotype	1	102.42	111.91	140.28	<0.0001
Mating	1	1.8380	11.329	2.5176	0.1366
Replicate	4	16.200	25.691	5.5474	0.0079

PC3 *Effect*	*Df*	*Sum of sq*	*RSS*	*F*	*P*
*Genotype*Mating*	1	2.0705	71.065	0.3601	0.5596
Genotype	1	4.3395	75.404	0.7938	0.3891
Mating	1	0.0131	71.078	0.0024	0.9616
Replicate	4	27.418	98.482	1.2539	0.3370

Table S2. SFPs detected as significantly differentially-abundant in response to genotype. q-values are calculated by applying a tail-based FDR correction to *p*-values obtained from a linear model iterated over each detected protein. q-values are given both for the effect of mating status (pre-mating/mated) and the genotype (Dad/control). Fold changes are given on a $\log_2$ scale and calculated for each genotype comparison within a mating status. Dad value is subtracted from the control. Therefore, positive values indicate greater abundance in controls. The transfer value is calculated by subtracting the Dad pre/post-mating fold change from the control pre/post-mating fold change. Therefore, positive values indicate greater transfer of an SFP to females in controls. Functional information associated with each protein's FlyBase entry is provided.

	qval		Log fold change				
Protein	Genotype	Mating	Virgin	Mated	Transfer	Functional class	Predicted function
Acp26Ab	0.0001	0.0252	-3.9921	-2.2305	-1.7616	Peptide/Prohormone	Post-mating behavior
antr	0.0099	0.0001	-0.4067	-0.4717	0.0650	CRISP	Defence response
CG11598	0.0001	0.0001	-0.8491	-0.8736	0.0245	Acid lipase	Lipase activity
CG31413	0.0021	0.4695	0.5486	0.6916	-0.1430	Thioredoxin	Protein modification process
CG6690	0.0204	0.0001	0.5501	0.4407	0.1094	Thioredoxin	Protein modification process
CG9997	0.0063	0.0001	-0.4432	-0.5971	0.1539	Serine protease	Proteolysis
NLaz	0.0001	0.0163	1.3765	1.3397	0.0368	Lipocalin	Lipid metabolic process
Sfp24C1	0.0001	0.0001	0.9107	0.7763	0.1344	Serine protease	Endopeptidase inhibitor
Spn28F	0.0011	0.0001	-0.6338	-0.7392	0.1054	Serpin	Negative regulation of proteolysis
Spn77Bb	0.0003	0.0001	-0.6584	-0.8108	0.1524	Serpin	Negative regulation of proteolysis
Spn77Bc	0.0363	0.0002	-0.5505	-0.4633	-0.0871	Serpin	Negative regulation of proteolysis

Table S3. Non-SFPs detected as significantly differentially-abundant in response to genotype. q-values are calculated by applying a tail-based FDR correction to *p*-values obtained from a linear model iterated over each detected protein. q-values are given both for the effect of mating status (pre-mating/mated) and the genotype (Dad/control). Fold changes are given on a $\log_2$ scale and calculated for each genotype comparison within a mating status. Dad value is subtracted from the control. Therefore, positive values indicate greater abundance in controls. Functional information associated with each protein's FlyBase entry is provided.

		qval		Log fold change			
Protein	Synonym	Genotype	Mating	Virgin	Mated	Functional class	Predicted function
34F4T	CG6084	0.0144	0.5982	-0.5304	-0.2470	Aldo-keto reductase	Oxidoreductase activity
CG8628		0.0234	0.5915	-1.3997	-0.9496	Acyl-CoA binding	Fatty-acyl-CoA binding
Arc1	CG12505	0.0145	0.3881	0.7358	0.6966	ARC/ARG3.1 family	mRNA binding, vesicle transport
Ars2	CG7843	0.0146	0.0402	-0.5872	-0.4427	Protein binding	RNA processing
BP1025	CG34002	0.0381	0.0001	-0.2350	-0.3303	CRISP	
CCT3	CG8977	0.0003	0.0001	0.5754	0.5030	TCP-1 chaperonin	ATP-binding
CG12262	Mcad	0.0292	0.4068	0.1925	0.1962	Acyl-CoA dehydrogenase	Acyl-CoA dehydrogenase activity
CG1532		0.0185	0.0001	-0.2595	-0.2447		
CG34034	BP1088	0.0006	0.0001	0.9092	0.7807		Ejacualtory duct protein
CG7408		0.0212	0.2767	0.4288	0.3982	Sulfatase	Sulfuric ester hydrolase activity
CG7966		0.0002	0.6415	-1.4620	-1.5465		Selenium binding
CG8303		0.0197	0.6473	-0.3672	-0.4004	Acyl-CoA binding	Fatty-acyl-CoA binding
Chd64	CG14996	0.0350	0.6093	-0.3380	-0.1718	Actin binding	Muscle contraction; JH response element
coro	CG9446	0.0483	0.0022	-0.2318	-0.1948		Somatic muscle development
Cpr	CG11567	0.0189	0.0670	0.2174	0.2890	NADPH cytochrome P450 reductase	Oxidoreductase activity
GH26	CG6287	0.0128	0.1044	-0.2145	-0.1897	Oxidoreductase	NAD binding
GLaz	CG4604	0.0339	0.0001	-0.2112	-0.8633	Lipocalin	Fatty acid binding
Hsp23	CG4463	0.0274	0.5398	2.1436	1.8039	Small heat shock protein	Protein folding
Hsp27	CG4466	0.0016	0.6320	10.3680	12.0168	Small heat shock protein	Protein folding
Idh	CG7176	0.0466	0.4891	0.2491	0.2592	Oxidoreductase	Isocitrate dehydrogenase activity
Lsp2	CG6806	0.0237	0.5855	-0.3754	-0.5225	Hemocyanin	Amino acid storage
Ncc69	CG4357	0.0192	0.0065	-0.4123	-0.4141	Chloride transporter	Transmembrane transporter activity
NTPase	CG3059	0.0127	0.0244	-0.2493	-0.2697	Nucleoside phosphatase	Diphosphatase activity
PPO1	CG42639	0.0004	0.0939	-0.7474	-0.7863	Phenoloxidase	Immune/Melanization
sphinx2	CG32382	0.0140	0.0008	-0.7809	-0.9257	Serine protease homolog	Proteolysis
tweek	CG42555	0.0001	0.1373	2.5610	1.9503	Fragile site associated protein	Vesicle endocytosis
